# Supplementary material for: Identification and functional analysis of non-coding regulatory small RNA FenSr3 in Bacillus amyloliquefaciens LPB-18
Source: PeerJ. 2023 May 15;11:e15236. doi: 10.7717/peerj.15236 (PMC10194069; doi:10.7717/peerj.15236)
Supplement: Supplemental Information 4 [file peerj-11-15236-s004.zip › KO/CK-vs-T1_map/map04934.html]

KEGG PATHWAY: Cushing syndrome - Reference pathway


|  |  |
| --- | --- |
| **Cushing syndrome - Reference pathway** |  |

[
Pathway menu
| Organism menu
| Pathway entry
| Show description
| User data mapping
]

Cushing syndrome (CS) is a rare disorder resulting from prolonged exposure to excess glucocorticoids via exogenous and endogenous sources. The typical clinical features of CS are related to hypercortisolism and include accumulation of central fat, moon facies, neuromuscular weakness, osteoporosis or bone fractures, metabolic complications, and mood changes. Traditionally, endogenous CS is classified as adrenocorticotropic hormone (ACTH)-dependent (about 80%) or ACTH- independent (about 20%). Among ACTH-dependent forms, pituitary corticotroph adenoma (Cushing's disease) is most common. Most pituitary tumors are sporadic, resulting from monoclonal expansion of a single mutated cell. Recently recurrent activating somatic driver mutations in the ubiquitin-specific protease 8 gene (USP8) were identified in almost half of corticotroph adenoma. Germline mutations in MEN1 (encoding menin), AIP (encoding aryl-hydrocarbon receptor-interacting protein), PRKAR1A (encoding cAMP-dependent protein kinase type I alpha regulatory subunit) and CDKN1B (encoding cyclin-dependent kinase inhibitor 1B; also known as p27 Kip1) have been identified in familial forms of pituitary adenomas. However, the frequency of familial pituitary adenomas is less than 5% in patients with pituitary adenomas. Among ACTH-independent CS, adrenal adenoma is most common. Rare adrenal causes of CS include primary bilateral macronodular adrenal hyperplasia (BMAH) or primary pigmented nodular adrenocortical disease (PPNAD).

|  |  |  |
| --- | --- | --- |
| Reference pathway | 184% 150% 122% 100% 82% 67% 55% | 图片下载 |
